# Effect of orofacial myofunctional therapy along with preformed appliances on patients with mixed dentition and lip incompetence

**DOI:** 10.1186/s12903-022-02645-w

**Published:** 2022-12-09

**Authors:** Xue Yang, Guangyun Lai, Jun Wang

**Affiliations:** 1 Department of Pediatric Dentistry, Shanghai Ninth People’s Hospital, Shanghai Jiao Tong University School of Medicine, Shanghai, China; 2 College of Stomatology, Shanghai Jiao Tong University, Shanghai, China; 3 National Center for Stomatology, Shanghai, China; 4 National Clinical Research Center for Oral Diseases, Shanghai, China; 5 Shanghai Key Laboratory of Stomatology, Shanghai, China; 6 Shanghai Research Institute of Stomotology, Shanghai, China

**Keywords:** Orofacial myofunctional therapy (OMT), Mixed dentition, Malocclusion correction, Efficacy, Lip strength

## Abstract

**Background:**

Various preformed early orthodontic appliances for correcting oral habits and training orofacial muscles have emerged on the market. However, there are few reports on the effectiveness of these appliances for orofacial myofunctional training.

**Methods:**

This retrospective study evaluated children with lip incompetence and mixed dentition treated at the Pediatric Dentistry Department of Shanghai Ninth People’s Hospital from 2016 to 2018. A total of 109 children (54 males, 55 females; age range: 7–10 years, mean age: 8.2 years) were selected from an overall sample of 870 patients. During the first visit, all patients were instructed to perform active lip and tongue training at home daily and were divided into two groups according to the kind of appliances worn. The first group consisted of 56 subjects (30 females; 26 males), with a mean age of 8.1 years (SD 1.1 years), treated with preformed appliances. The second group consisted of 53 subjects (25 females; 28 males), with a mean age of 8.2 years (SD 1.0 years), treated with conventional early orthodontic appliances (arch expansion devices along with "2*4" local fixed appliances). For each subject in the two groups, initial (pretreatment, T1) and final (posttreatment, T2) intraoral and external photos, dental casts, lateral cephalograms, and orthopantograms were taken, and lip strength was measured. SNA, SNB, ANB, APDI, FMA, U1SN, and IMPA before and after treatment were measured by The Dolphin Imaging Cephalometric Analysis Software. The hyoid bone position was also recorded. Differences between groups were identified with an independent sample t-test (*P* < 0.05).

**Results:**

In the first group, a statistically significant forward movement of the mandible was detected by an increase in SNB of − 1.06 degrees (*P* < 0.01) and an increase in APDI of − 2.23 degrees (*P* < 0.01). The increase in IMPA (− 3.21 degrees, *P* < 0.01) demonstrated a statistically significant protrusion of the lower incisors. Lip strength significantly increased (− 2.44*, P* < 0.01). The increase in HC3 (− 1 mm, *P* < 0.01) and HFH (− 2.95 mm, *P* < 0.01) implied a forward and downward movement of the hyoid bone. In the second group, a statistically significant forward movement of the mandible was also detected by an increase in APDI of -1.96 degrees (*P* < 0.01). Lip strength also significantly increased (− 1.24*, P* < 0.01). The increase in HFH (− 2.55 mm, *P* < 0.01) implied a downward movement of the hyoid bone. Compared with the treatment in the second group, orofacial myofunctional therapy combined with the preformed appliances led to a statistically significant lip strength increase (− 2.30, *P* < 0.05). Significant differences were observed in SNB and IMPA between the two groups (*P* < 0.05).

**Conclusions:**

Orofacial myofunctional therapy effectively improved patient lip strength and was a good option for mixed dentition patients with lip incompetence. Preformed appliances could enhance the orofacial myofunctional therapy effect and result in significant improvements in lip strength and forward movement of the mandible, which can optimize the jaw relationship.

## Introduction

In addition to the regular role in eating, the oral cavity assists with nasal breathing and pronunciation, which require the synergy of many muscles, such as those of the mouth, face, and neck. The relationships between functional behavior and dental development have been discussed for a long time. Previously, Angle realized that the function of the cheeks, tongue, and lips influenced the occurrence and persistence of malocclusions [1]. Besides genetic factors, physiological factors such as the morphology, position, and abnormal function of the orofacial muscles, lips, cheeks, tongue, and chewing muscles are also important.

The prevalence of malocclusion and orofacial dysfunctions in children and adolescents is high. Gabrowski *et.al.* found normal occlusal relationships in only 25.3% of children with primary dentition. The frequency of children with normal dentition significantly decreased among those with mixed dentition (7.3%) [2]. Franka *et.al.* reported that every fourth child with primary dentition and every third with mixed dentition presented three or more orofacial dysfunctions. The frequency rates of orofacial dysfunction in primary and mixed dentition were 61.6% and 80.8%, respectively [3]. Oral habits, which have been widely studied in the literature, are thought to influence dental development. 62.0% of children with primary dentition and 63.5% of children with mixed dentition showed atypical swallowing respectively, which was the most frequently diagnosed orofacial dysfunction. Additionally, habitual open mouth posture was found in 37.3% of children with primary dentition and 42.0% of those with mixed dentition [3].

Moreover, it was reported that compared with children with normal dentition, children with frontal open bite, lateral crossbite, and increased overjet tended to present static functional disturbances, such as the open mouth and compensatory tongue postures. Postural anomalies and postural habits were most likely to significantly influence the development of dental occlusion in all three dimensions [4].

Theories regarding the growth of the facial skull emphasize the influence of function. The stability of the final position of the teeth depends on the harmony of the oral and maxillofacial system and the respiratory system. An imbalance in neuromuscular forces leads to malocclusion [5]. The orthodontic treatment outcome in the presence of muscle dysfunction is unstable. Therefore, clinicians need to correct orofacial dysfunctions as soon as possible.

Orofacial myofunctional therapy (OMT) aims to establish a new neuromuscular pattern and correct abnormal functional and resting postures. It can strengthen the orofacial muscles to pave the way for mouth closure at rest, establishing nasal breathing, and learning a physiological swallowing pattern [6]. In addition to OMT, various preformed early orthodontic appliances are on the market for oral habits correction and orofacial muscle training. However, reports on the effectiveness of these dental appliances on orofacial myofunctional training are rare. Therefore, in this study, lip strength, hyoid position, and early orthodontic effect were quantitatively measured to evaluate the effect of orofacial myofunctional training on correcting middle-mixed dentition malocclusions. We hope this study can help guide clinical treatment, improve early orthodontic outcomes, and provide the theoretical basis for large-scale clinical randomized controlled trials.

## Materials and methods

### Ethical consideration

This project was performed in accordance with the Declaration of Helsinki and was approved by the Ethics Committee of Shanghai Ninth People’s Hospital, Shanghai Jiao Tong University School of Medicine (No. SH9H-2021-T332-1).

### Subject grouping and treatment modalities

Patients with lip incompetence and mixed dentition were selected from all patients treated at the Pediatric Dentistry Department of Shanghai Ninth People’s Hospital from 2016 to 2018. All patients in the final sample were included according to the criteria listed in Table 1.

**Table 1:** Inclusion and exclusion criteria

*Inclusion criteria*
Eight incisors have been completely replaced, and primary canines and primary molars have not started to be replaced
Angle class I or Class II
Incompetent lips
One or more of the following features: ① Crowding: ≤ 4 mm; ② Anterior teeth overjet I°-II°; and ③ Anterior teeth deep overbite
*Exclusion criteria*
Angle class III
Severe skeletal class II
Moderate to severe crowding
High angle, open bite tendency and skeletal deviation
Poor compliance and coordination
Adenoid, tonsil hypertrophy with indications for resection but no surgery for an obstructed nasal airway
Allergy to oral materials

Finally, 109 children (54 males, 55 females; age range: 7–10 years, mean age: 8.2 years) were selected from an overall sample of 870 patients. All the selected children were divided into two groups according to the kind of appliances worn. The first group consisted of 56 subjects (30 females; 26 males), with a mean age of 8.1 years (SD 1.1 years), treated with preformed appliances (MRC Myofunctional Research Co. Queensland, Australia). The second group consisted of 53 subjects (25 females; 28 males), with a mean age of 8.2 years (SD 1.0 years), treated with conventional early orthodontic appliances (arch expansion devices along with "2*4" local fixed appliances). The two groups had no significant differences in sex, average age, or primary lip strength.

At the first visit, all the children were instructed to perform active lip and tongue training daily, which was supervised and recorded by their parents at home. (First, lip muscle training: 1. Lip closure and competency exercise: tightly closing the lips together, holding a piece of cardboard/popsicle stick between the upper and lower lip for 1 h a day. 2. "lip kisses" 100 times a day. Second, tongue muscle training: chewing gum was placed on the tip of the tongue, and then the tip of the tongue was placed against the palate right behind the upper front teeth and pressed down on the gum while swallowing was performed with the teeth closed for 10 min a day.)

Patients in the first group used preformed appliances (MRC Myofunctional Research Co. Queensland, Australia) at night (≥ 8 h) during sleep and continuously for 2 h during the day. The patients visited dentists monthly, and next-stage appliances were interchanged in time until the anterior teeth alignment was achieved. The mean duration of treatment was 354 days.

Patients in the second group used conventional early orthodontic appliances (removable expansion devices and "2*4" local fixed appliances). The treatment continued until the anterior teeth alignment was achieved. The mean duration of treatment was 322 days.

### Evaluation of treatment outcomes

For each subject in the two groups, internal and external photos, dental casts, lateral cephalograms, orthopantograms (OPGs), and lip strength before (T1) and after (T2) treatment were collected.

**Fig. 1:**
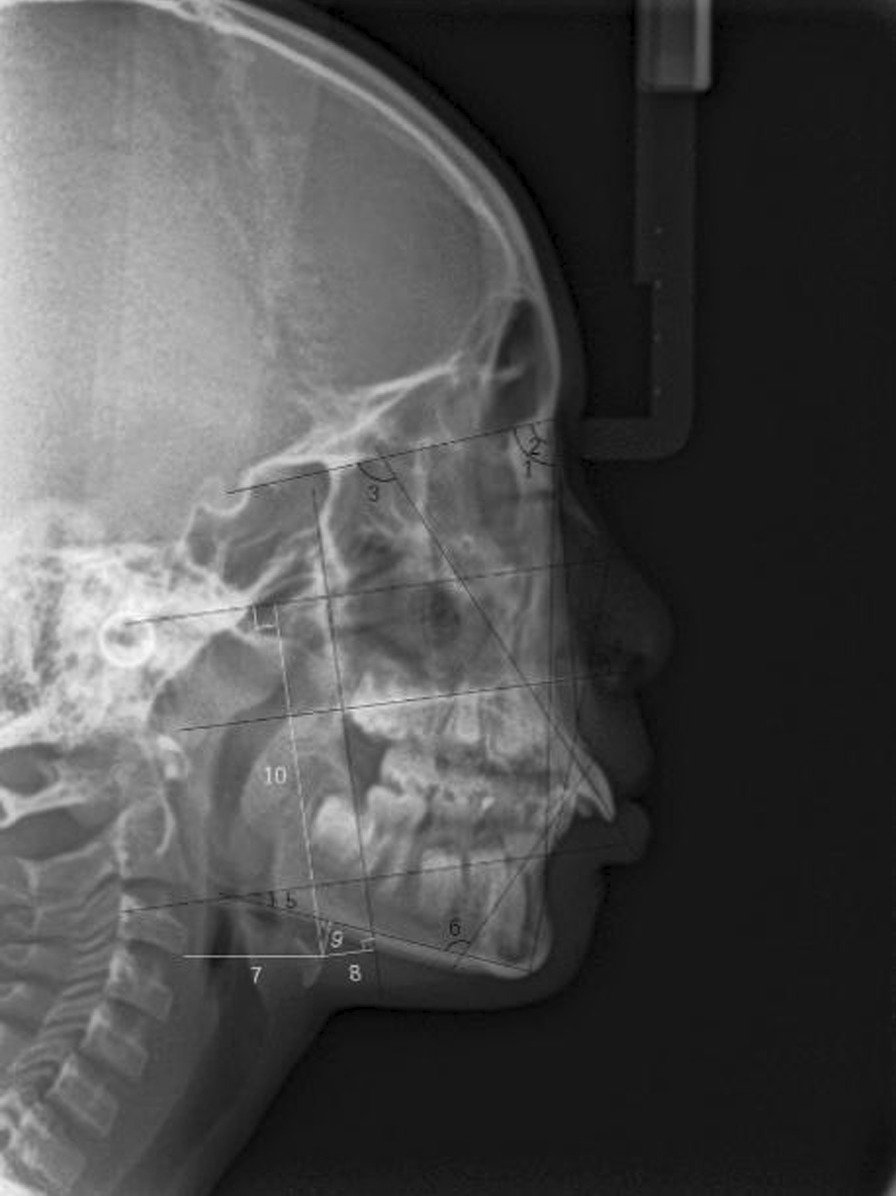
The position of the hyoid bone

For each patient, lateral cephalograms were taken before and after treatment in the Department of Oral Radiology, Shanghai Ninth People’s Hospital. SNA, SNB, ANB, APDI, FMA, U1SN, and IMPA before and after treatment were measured by Dolphin Imaging Cephalometric Analysis Software (Dolphin, America). The position of the hyoid bone was described and recorded (Table 2 and Fig. 1).

**Table 2:** The position of the hyoid bone

*Horizontal position*
HC3: The distance from the anterior upper point of the hyoid bone (H) to the lower edge of the third cervical vertebra (C3)
HPtm: The distance from H to the vertical line of the FH through the Ptm point
*Vertical position*
HFH: The distance from H to the FH plane
HMP: The distance from H to the MP plane (Go-Gn)

### Data collection and statistical analysis

To determine methodological error, the cephalometric radiographs were traced and measured by one trained examiner who repeated the analysis after an interval of approximately one week. Cohen’s kappa was used to compare the two measurements (systematic error).

Descriptive statistics were calculated for all measurements in each group. A paired t-test was performed to compare the measurement items in each group. Significant between-group differences were identified with an independent sample t-test. *P* < 0.05 indicated that the difference was statistically significant. The means and standard deviations of the measurements are presented. All the data were analyzed using the Statistical Package for Social Sciences version 25.0 (SPSS Inc., Chicago, Illinois, USA).

## Results

No systematic error was found between the repeated measurements.

Statistical comparisons between the boys and girls in each group after treatment were shown in Tables 3 and 4.

**Table 3:** Statistical comparison between the boys and girls in the first group

	Sex	T1–T2		*t* value	*P* value
		Mean	SD		
LS	1	–2.29	2.447	0.450	0.655
	2	–2.59	2.631		
SNA (°)	1	–0.22	1.695	0.721	0.192
	2	–0.41	2.244		
SNB (°)	1	–1.00	1.330	0.778	0.121
	2	–1.12	1.801		
FMA (°)	1	–0.48	2.045	0.308	–0.585
	2	0.10	2.193		
APDI (°)	1	–2.19	2.512	0.912	0.091
	2	–2.28	3.473		
U1SN (°)	1	2.11	7.733	0.641	0.835
	2	1.28	5.483		
IMPA (°)	1	–3.52	5.102	0.672	–0.585
	2	–2.93	5.230		
HC3 (mm)	1	–1.30	2.308	0.464	–0.564
	2	–0.73	3.292		
HPtm (mm)	1	2.09	5.376	0.240	1.708
	2	0.39	5.375		
HFH (mm)	1	–2.25	4.687	0.332	1.349
	2	–3.60	5.546		
HMP (mm)	1	1.69	5.273	0.120	2.032
	2	–0.34	4.345		

Sex: 1 boy, 2 girl

*SD* standard deviation

**Table 4:** Statistical comparison between the boys and girls in the second group

	Sex	T1–T2		*t* value	*P* value
		Mean	SD		
LS	1	–0.97	2.922	0.689	0.494
	2	–1.53	2.999		
SNA (°)	1	–0.11	1.988	–0.529	0.599
	2	0.16	1.650		
SNB (°)	1	–0.29	1.652	0.739	0.463
	2	–0.60	1.443		
FMA (°)	1	0.14	2.940	0.360	0.721
	2	–0.12	2.297		
APDI (°)	1	–1.68	2.144	1.036	0.305
	2	–2.28	2.072		
U1SN (°)	1	0.84	9.627	–0.615	0.541
	2	2.20	5.759		
IMPA (°)	1	0.48	6.917	–1.433	0.158
	2	2.76	4.136		
HC3 (mm)	1	–0.69	2.565	–0.930	0.357
	2	0.06	3.333		
HPtm (mm)	1	0.50	5.105	0.382	0.704
	2	–0.05	5.269		
HFH (mm)	1	–1.66	6.231	1.170	0.247
	2	–3.55	5.444		
HMP (mm)	1	–0.11	5.532	0.988	0.328
	2	–1.42	3.835		

Sex: 1 boy, 2 girl

*SD* standard deviation

The cephalometric variables, lip strength data at T1 and T2 and the differences between the two groups were shown in Table 5.

**Table 5:** Variables before (T1) and after (T2) treatment between the two groups

	Group	T1		T2		T1–T2			*t* value	*P2* value
		Mean	SD	Mean	SD	Mean	SD	*P1* value		
LS	1	6.67	1.80	9.12	2.69	–2.44	2.53	0.00**	–2.30	0.02*
	2	7.26	2.21	8.50	2.66	–1.24	2.94	0.00**		
SNA (°)	1	81.09	3.08	81.41	3.12	–0.32	1.98	0.23	–0.93	0.35
	2	81.09	3.60	81.08	3.68	0.02	1.82	0.94		
SNB (°)	1	74.88	3.00	75.95	3.19	–1.06	1.58	0.00**	–2.10	0.04*
	2	75.81	3.62	76.25	3.40	–0.43	1.55	0.05		
FMA (°)	1	27.95	4.13	28.13	4.31	–0.18	2.12	0.53	–0.43	0.67
	2	29.34	4.65	29.32	5.03	0.02	2.64	0.96		
APDI (°)	1	74.07	3.86	76.30	3.88	–2.23	3.02	0.00**	–0.54	0.59
	2	76.30	4.63	78.26	4.77	–1.96	2.11	0.00**		
U1SN (°)	1	105.27	10.30	103.59	7.72	1.68	6.61	0.06	0.14	0.89
	2	106.93	8.21	105.45	6.88	1.48	7.99	0.18		
IMPA (°)	1	93.89	6.84	97.11	7.07	–3.21	5.13	0.00**	–4.54	0.00**
	2	94.20	5.64	92.64	6.06	1.56	5.84	0.06		
HC3 (mm)	1	28.63	2.68	29.63	3.11	–1.00	2.85	0.01*	–1.20	0.23
	2	28.76	2.87	29.10	3.03	–0.34	2.95	0.41		
HPtm (mm)	1	13.88	6.29	12.67	7.30	1.21	5.40	0.09	0.96	0.34
	2	9.46	7.18	9.22	6.56	0.24	5.14	0.73		
HFH (mm)	1	79.74	10.33	82.69	9.62	–2.95	5.15	0.00**	0.38	0.71
	2	72.45	9.68	75.00	9.51	–2.55	5.89	0.00**		
HMP (mm)	1	10.81	5.28	10.17	4.69	0.64	4.88	0.33	1.48	0.14
	2	9.27	4.98	9.99	4.70	–0.73	4.81	0.27		

*SD* standard deviation, *P1* paired t-test within each group, *P2* independent t-test between two groups

*P < 0.05, **P < 0.01

No statistically significant differences in the measurements after treatment were found between the boys and girls in each group (Tables 3 and 4).

The cephalometric measurements in the first group revealed significant forward movement of the mandible, as indicated by an increase in SNB of -1.06 degrees (*P* < 0.01) and an increase in APDI of − 2.23 degrees (*P* < 0.01). The increase in IMPA (− 3.21 degrees, *P* < 0.01) indicated a significant protrusion of the lower incisors. Forward and downward movement of the hyoid bone was evidenced by an increase in HC3 (− 1 mm, *P* < 0.05) and HFH (− 2.95 mm, *P* < 0.01). U1SN slightly decreased, and FMA slightly increased, but no statistically significant changes were found (*P* > 0.05).

In the second group, a significant forward movement of the mandible was also observed by an increase in APDI of − 1.96 degrees (*P* < 0.01). A significant increase in lip strength (− 1.24*, P* < 0.01) was also detected. The increase in HFH (− 2.55 mm, *P* < 0.01) indicated a downward movement of the hyoid bone.

An independent sample t-test was performed on the data of the two groups before and after the treatment. Compared with the treatment in the second group, OMT combined with the preformed appliances showed a statistically significant increase in lip strength (− 2.30, *P* < 0.05). The cephalometric measurements in the first group revealed a significant forward movement of the mandible, as indicated by an increase in SNB of − 2.1 degrees (*P* < 0.05). Patients in the first group who used the preformed appliances showed greater lower incisor protrusion (− 4.54 degrees, *P* < 0.01) than those in the second group. However, there was no significant difference in the position of the hyoid bone between the two groups after treatment (*P* > 0.05) (Table 5).

## Discussion

### The relationships among perioral muscles, the position of the anterior teeth and malocclusion

Abnormal shape, position and function of the lip and tongue as well as other perioral muscles are critical causes of malocclusion. Cattoni [7] demonstrated that the lips were naturally closed during normal nasal breathing, the tongue was lifted, and the maxillofacial region was well developed. Furthermore, abnormal airflow entered the oral cavity in patients who breathe for a long time through their mouths. The upper lip was short and upturned. The protruded upper anterior teeth, the lowered tongue, and the narrow upper dental arch further aggravated the protrusion of the upper anterior teeth. Insufficient upper lip muscle strength could easily lead to lip incompetence. Hassan [8] showed that the strength of the labial muscles was closely related to the position of the anterior teeth. Burstone [9] found that the muscle strength of the lower lip played an essential role in maintaining the normal position of the upper and lower anterior teeth at rest. When the pressure on the lower lip muscle was too high, retrusion of the lower incisors, mandible retraction, and Angle Class II division 2 malocclusion appeared. When the lower lip muscle strength was too weak, protrusion of the upper incisors would appear.

Rogers [10] reported that the perioral muscle functional training method could correct malformations and deformities. He believed that the perioral muscles could be changed after training, thus affecting the position of the teeth and changing the shape of the dental arch.

In this study, through the active muscle functional training method, the lip strength of 109 children significantly increased after treatment, as indicated by an increase in LS in the first group (− 2.44, *P* < 0.01) and second group (− 1.24, *P* < 0.01). The difference between the two groups was significant, which might be attributed to the effect of the preformed appliance. Compared with the children in the second group, patients in the first group showed more mandibular advancement, which can benefit maxillofacial development and profile improvement. This result was consistent with the study by Usumez [11]. The mandible of the second group also moved forward after treatment, which may be related to the expansion of the upper dental arch and improved alignment of the anterior teeth after treatment.

### Lip strength measurement

In the 1970s, Posen described a method of measuring the strength of the lips for clinical use [12]. Over the past decades, many different measurement systems for lip strength have been developed. These were roughly classified into three main types: (1) tension gauge type. (2) balloon type. (3) strain gauge type [13]. These systems, however, were challenging to operate and use clinically, especially for children. Saitoh reported that lip strength in children had two different stages: one was a period of development (3–6 years old), and the other was a stable period (7–12 years old) [13]. In our study, the lip strength before and after treatment was collected by a digital medical strain gauge (Lipplekun; Shofu, Kyoto, Japan), which has been proven reliable [14]. In our study, the age range of the patients was 7 to 10 years old, indicating that they were in a stable period. The significant improvement in lip strength was mainly due to muscle function training.

### Relationships among abnormal tongue position, hyoid bone position and malocclusion

Urzal [15] and others believed that the tongue could exert a force up to 500 g, while less than 2 g was enough to move the incisors. At rest, the tip of the tongue should be located at the incisor papilla 5 mm behind the upper incisors, and the back of the tongue should be held against the palate. The pressure exerted by the tongue was one of the main factors in maintaining the position of teeth. The lateral force exerted by the tongue promoted maxillary dental arch width development. The balance of labio-lingual muscle strength determined the position of the anterior teeth, while the balance of buccal-lingual muscle strength determined the position of the posterior teeth. Mason [16] pointed out that posture at rest was more important than the functional position of the tongue. An abnormal tongue position at rest could easily lead to a malocclusion such as upper dental arch stenosis. Establishing the correct resting tongue position was beneficial for stabilizing the outcome after correcting malocclusion and preventing relapse.

An abnormal dynamic tongue position mainly refers to abnormal swallowing, in which the tongue extends forward to form a seal with the lip or extends out of the mouth for swallowing. Abnormal swallowing can be a simple tongue thrust or tongue thrust combined with compensatory muscle recruitment. In tongue thrust, the tongue extends between the upper and lower teeth with force continuously acting on the teeth. The tongue thrust affects the normal eruption of the teeth, which can cause open bite deformity. Rogers [10] proposed that tongue thrust, combined with excessive contraction of the labial muscle, buccal muscle and mental muscle, easily led to a narrow upper arch, upper incisor protrusion, lingual inclination of the lower incisors, mandibular retraction and deep overjet of the anterior teeth.

The hyoid bone is a vital structure around the body of the tongue. The body of the tongue is attached to the hyoid bone through ligaments and muscles such as the hyoglossus muscle, which are independent and connected with each other [17]. The hyoid bone position affects the tongue body's position in the oral cavity, and an abnormal position of the hyoid bone also accompanies an abnormal position of the tongue. Haralabakis [18] found that the position of the hyoid bone was closely related to the tongue position, and the position of the hyoid bone in open bite patients was relatively higher than that in patients with a normal bite. Gokce and other scholars [19] found a specific abnormal position of the hyoid bone in patients with skeletal Class III malocclusion, which was closely related to abnormal swallowing activities. There was a relationship between mandibular positional changes and the position of the hyoid bone [20]. The position of the hyoid bone in Class III malocclusion patients was lower than that in Class I patients [21].

In this study, the position of the mandible moved forward, the jaw relationship improved, and the hyoid bone moved forward and downward, with statistically significant differences in the first group. This result was consistent with the previous studies, in which the position of the hyoid bone moved forward and downward after treatment with functional appliances for patients with class II division 1 malocclusion [22, 23]. In the second group, the mandible also moved forward after treatment, which may be related to the expansion of the upper dental arch and the alignment of the anterior teeth after treatment. The increase in HFH (− 2.55 mm, *P* < 0.01) implied a downward movement of the hyoid bone in the second group that might be attributed to the muscle functional training efforts.

### Orofacial myofunctional therapy

Orofacial myofunctional therapy assesses, diagnoses, and treats patients with abnormal orofacial muscle function [24]. Through re-education of the nerves and muscles of the oral and maxillofacial region, the oral habits can be corrected, while the normal development of the craniofacial structure and the coordination and stability of the oral and maxillofacial system functions can be promoted. However, good patient and parental compliance are keys to the treatment of orofacial muscle function, and the training takes time and practice to be effective. In addition, it is crucial to control the indications and contraindications for OMT. This approach has limited effects in treating severe skeletal malocclusion, open bites, and severe tooth and bone volume irregularity. Conventional orthodontics and even the combination of orthognathic surgery and orthodontics are still required for the treatment of permanent teeth. This study investigated the hyoid bone position. A comparison of tongue position can be added in a future study to evaluate the comprehensive effect of OMT more accurately.

## Conclusions

Orofacial myofunctional therapy effectively improved the patient’s lip strength and was a good option for mixed dentition patients with lip incompetence. Preformed appliances could enhance the OMT effect and result in significant improvement in lip strength and forward movement of the mandible, which can optimize the jaw relationship. However, lower incisor protrusion was evident after treatment, suggesting that more attention should be given to the labial gingiva of the lower anterior teeth during treatment.

## Data Availability

The dataset used in this article is available from the corresponding author upon reasonable request.
